# Endoscopic resection of upper gastrointestinal lymphangioma: A single-center experience

**DOI:** 10.3389/fonc.2022.1030039

**Published:** 2022-11-09

**Authors:** Ziyu Shi, Xue Huang, Keliang Li, Qiuyue Tu, Dan Liu, Lixia Zhao, Huiyu Yang, Deliang Li, Yue Zhao, Jiyu Zhang, Muhan Li, Bingrong Liu

**Affiliations:** ^1^ Department of Gastroenterology, The First Affiliated Hospital of Zhengzhou University, Zhengzhou, China; ^2^ Academy of Medical Science, Zhengzhou University, Zhengzhou, China

**Keywords:** endoscopic ultrasonography (EUS), en bloc resection, clinical outcomes, vascular tumor, upper gastrointestinal lymphangioma

## Abstract

**Objective:**

Lymphagioma, which in most cases as benign tumors, occurs in head, neck, axilla, and mediastinum. Lymphangioma is exceedingly rare in the upper gastrointestinal tract including esophagus, stomach, and duodenum. However, the clinical characteristics, natural history, and recurrence rate after endoscopic resection remain unclear. This study aims to evaluate the characteristic findings and assess the efficacy of endoscopic techniques in the management of this disease.

**Methods:**

In this systematic retrospective analysis, we evaluated all 24 cases of upper gastrointestinal lymphangioma resected by endoscopic mucosal resection (EMR) or endoscopic submucosal dissection (ESD) and diagnosed by histopathology at our hospital from January 2012 to May 2021. We analyzed the results of endoscopy, endoscopic ultrasonography (EUS), CT, histologic examination, and follow-up assessments.

**Results:**

9 male and 15 female patients with esophageal lymphangioma were enrolled in this study, with a mean age of 54.17 ± 11.60 years (range 30-71 years). The lesions’ size varied from 2.20 to 40.10 mm, with the median size of 7.83 mm. All patients were evaluated preoperatively, whose endoscopic appearance typically appears as dilated lymphatic channels beneath the surface epithelium of the protrude mucosal or sub-mucosal lesion. Endoscopic ultrasonography revealed the presence of a honeycomb-like or grid-like mass with a heterogeneous echo pattern, and a clear boundary between the lesion and the muscularis propria layer may be helpful for the primary diagnosis of this disease. 22 patients underwent EMR and 2 patient were treated with ESD. Histologic examination revealed that the lesions contained many dilated lymphatic vessels, which confirmed the initial diagnosis of lymphangioma in all patients. No major adverse events were found during the operation or a median follow-up of 43 months (range 13–92).

**Conclusions:**

Endoscopic ultrasonography has important clinical value for the primary diagnosis of lymphangioma in the upper gastrointestinal tract. This study also suggests that endoscopic resection should be considered as a more minimally invasive, safe, feasible, and effective therapeutic option comparing to laparoscopic surgery.

## Introduction

Described for the first time by Koch in 1913, lymphangiomas are benign congenital malformations of the lymphatic system and consist of dilated lymphatic vessels (1). As a type of tumor mostly appears as submucosal lesion, the incidence of lymphangiomas is 1.2–2.8%, and those tumors occur most frequently in head, neck, axilla, and mediastinum (1, 2). Lymphangiomas can be found at any age of life, of which approximately 50% are present at birth and 90% are diagnosed before the age of 2, and both genders are equally affected (3, 4). Within the gastrointestinal tract, the small bowel mesentery is the most common site involved, followed by retroperitoneal sites (5, 6). Lymphangiomas exceedingly rarely involve the upper gastrointestinal tract including esophagus, stomach, and duodenum (7, 8). These tumors are usually asymptomatic and found incidentally because the clinical presentation is highly polymorphic and nonspecific, contributing little to establish the diagnosis (9, 10). As a minimally invasive technique, endoscopic resection has come to play a pivotal role in the management of upper gastrointestinal lymphangioma (UGL) (11, 12). However, studies involving a mass of UGLs are lacking, and many questions need to be answered regarding the clinical characteristics, natural history and recurrence rate after resection (13). In this systematic retrospective analysis, we evaluated all cases of UGL diagnosed and resected endoscopically at our hospital from January 2012 to May 2021. We analyzed the results of endoscopy, endoscopic ultrasonography (EUS), computed tomography (CT), histologic examination, and follow-up assessments to clarify the characteristic findings and assess the efficacy of endoscopic techniques in the management of this disease as well as its related complications, diagnostic difficulties, and therapeutic problems.

## Materials and equipment

We report a systematic retrospective study from January 2012 to May 2021 concerning 24 patients who underwent EMR or ESD procedure for UGL in a tertiary hospital (The First Affiliated Hospital of Zhengzhou University, China). This retrospective study was approved by the Ethics Committee of this hospital.

The preparation, procedure, possible costs, and potential complications were explained to the patients or their family members in advance and all signed informed consent were provided by the participants. Before endoscopic treatment under general anesthesia, each patient had been evaluated by CT scans reviewed by two radiologists with more than 5-year experience. In addition, EUS was performed preoperatively in all 24 cases and each was reviewed by an experienced endoscopist who had performed more than 100 EUS examinations. Information about age, gender, clinical manifestation, CT scan results, lesion size, location, origin layer based on EUS, and complications was recorded.

The main endoscopic equipment and accessories include: Endoscopes (GIF-QF260J; Olympus Medical Science, Japan), Hook knife (KD-620LR; Olympus Medical Science, Tokyo, Japan), Insulation-tip knife (KD-611L; Olympus Medical Science, Tokyo, Japan), Snare with maximum insertion diameter of 1.8 mm (SD-221L-25; Olympus Medical Science, Japan), and Hemoclips (HXROCC-D-26-195-C, MICRO-TECH, China; HX-610-090 L, Olympus Medical Science, Tokyo, Japan).

## Methods

### Procedures

All patients were hospitalized and received general anesthesia during the operation. After the evaluation for patients, EMR or ESD was performed at the discretion of the endoscopist who will consider the preoperative examinations and intraoperative results. If the tumor was small and originated from the mucosal layer, the EMR was chosen. On the contrary, for large lymphangiomas originated from the submucosal layer without involving the muscle layer, the ESD was the superior option.

*EMR procedure* En bloc resection was defined as the tumor was excised in a whole piece, whose capsule was intact. First, a solution of adrenaline and saline was injected into the submucosal space under the lesion with the injection needle to prevent complications such as perforation and bleeding. Then a snare was inserted through the main channel to capture and fix the lesion which was then removed using electric coagulation. Finally, hemostasis was obtained with endoscopic electric coagulation.

*ESD procedure* First, circumferential marking was made using an insulated-tip knife at a distance of 0.5 to 1.0 cm from the lesion border. Then, a solution of adrenaline and saline was injected to lift the submucosa to enhance surgical safety as well as prevent further complications. After a circumferential mucosal incision was performed by endoscopic hook knife, the transparent cap assisted in stripping the lesion. Finally, the tumor was completely and uneventfully en bloc resected by foreign forceps and then hemostasis was obtained.

### Histopathological assessment

For all patients after the endoscopic resection procedure was performed, the specimens were stretched smoothly by pins on a corkboard and fixed with 10% formalin for later pathological examination. Every specimen was diagnosed by 2 expert gastrointestinal pathologists, providing a final confirmation.

### Follow-up

The evidence of postoperative complications including fever, dyspnea, hematemesis, and chest or abdominal pain was recorded. Each of the patients was discharged successfully and they were followed up by endoscopy and/or detailed telephone interviews. The interview’s outline was clinical symptoms, outcomes of treatment, and tests performed at their local hospitals.

### Statistical analysis

The statistical analyses were performed using IBM SPSS Statistics v23.0 (Statistical Package for the Social Sciences, Inc, Chicago, IL, United States). The mean ± SD was done as quantitative variables and percentage (%) as qualitative variables.

## Results

### Baseline characteristics

The data from the clinical assessment, treatment, and follow-up were analyzed retrospectively. From January 2012 to May 2021, more than 100 patients were diagnosed with Upper Gastrointestinal Lymphangioma by histopathological examination, 24 patients of whom underwent EMR or ESD procedures for UGL in the First Affiliated Hospital of Zhengzhou University. The average age at diagnosis was 54.17 ± 11.60 years (range 30-71 years). There were 9 (37.50%) males and 15 (62.50%) females with a sex ratio equal to 0.6. Clinically, the presentation was highly polymorphic. Most patients presented with multiple symptoms including abdominal pain (n = 9), abdominal discomfort (n = 5), regurgitation (n = 5), bloating (n = 3), foreign body sensation (n = 2). The main symptom for esophageal lymphangioma was abdominal discomfort (n = 4), while that for duodenal and gastric lymphangioma was abdominal pain (n = 6). Lymphangiectasia was histologically confirmed in one patient seven months before the endoscopic resection of the lymphangioma. The baseline characteristics of the 24 patients are shown in **Table 1**.

**Table 1:** Baseline characteristics.

Case	Gender	Age	Site	Size (mm)	Chief Complains	Endoscopic Appearance	Treatment	Follow-up time (M)
1	Female	58	Esophagus	5.7	Bloating	Submucosal mass	EMR	92
2	Female	47	Esophagus	10	Bloating	Nodule	EMR	73
3	Female	49	Esophagus	5.3	Abdominal pain	Mucosal prominence	EMR	67
4	Female	62	Esophagus	5	Abdominal pain	Protuberance	EMR	58
5	Female	41	Esophagus	3.9	Abdominal discomfort	Hemispherical protuberance	EMR	56
6	Male	62	Esophagus	6	Regurgitation	Mucosal prominence	EMR	48
7	Male	71	Esophagus	6.7	Abdominal pain	Submucosal mass	EMR	46
8	Female	61	Esophagus	6.2	Abdominal discomfort	Protuberance	EMR	45
9	Female	53	Esophagus	2.2	Abdominal discomfort	Submucosal mass	EMR	44
10	Male	65	Esophagus	5	Foreign body sensation	Submucosal mass	EMR	40
11	Male	61	Esophagus	7	Regurgitation	Hemispherical protuberance	EMR	34
12	Male	30	Esophagus	5.1	Abdominal discomfort	Submucosal mass	EMR	33
13	Male	56	Esophagus	4.8	Regurgitation	Nodule	EMR	29
14	Female	42	Esophagus	40	Foreign body sensation	Submucosal mass	ESD	26
15	Male	69	Duodenum	6.8	Abdominal pain	Nodule	EMR	55
16	Female	55	Duodenum	6.1	Abdominal pain	White protuberance	EMR	52
17	Male	60	Duodenum	3	Regurgitation	White diminutive nodule	EMR	51
18	Female	64	Duodenum	3	Regurgitation	Yellowish nodule with white spots	EMR	45
19	Female	69	Duodenum	6	Abdominal pain	Submucosal mass	EMR	33
20	Female	31	Duodenum	8	Abdominal pain	Yellowish submucosal mass	EMR	32
21	Female	33	Duodenum	5	Abdominal discomfort	Mucosal prominence with white spots	EMR	26
22	Female	48	Duodenum	10	Abdominal pain	Submucosal mass with white spots	EMR	20
23	Female	58	Duodenum	22.9	Bloating	Protuberance with white spots	ESD	13
24	Male	55	Stomach	4.1	Abdominal pain	Nodule	EMR	16

### Imaging features

**Figure 1:**
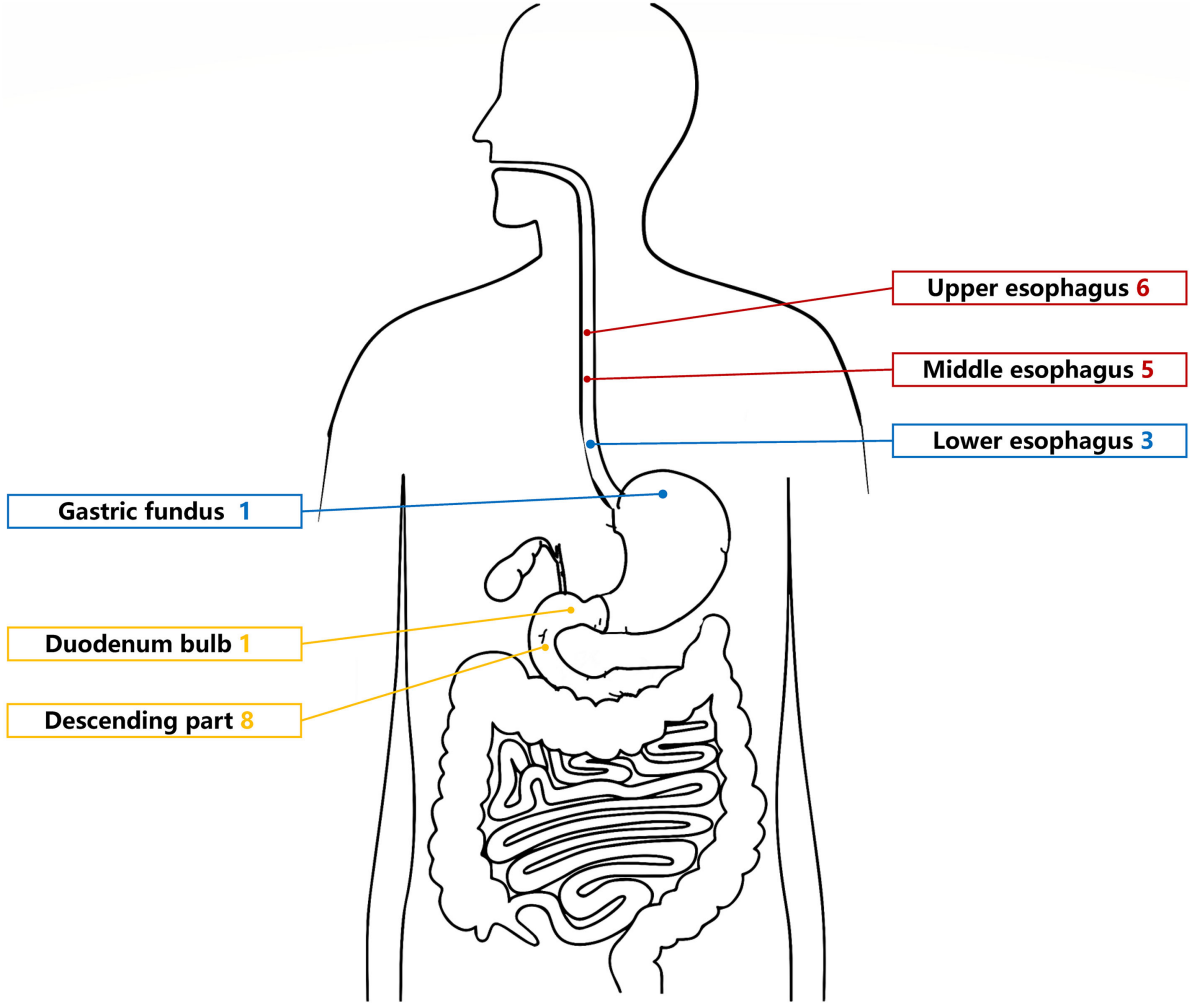
Site of lymphangiomas in the upper gastrointestinal tract.

**Figure 2:**
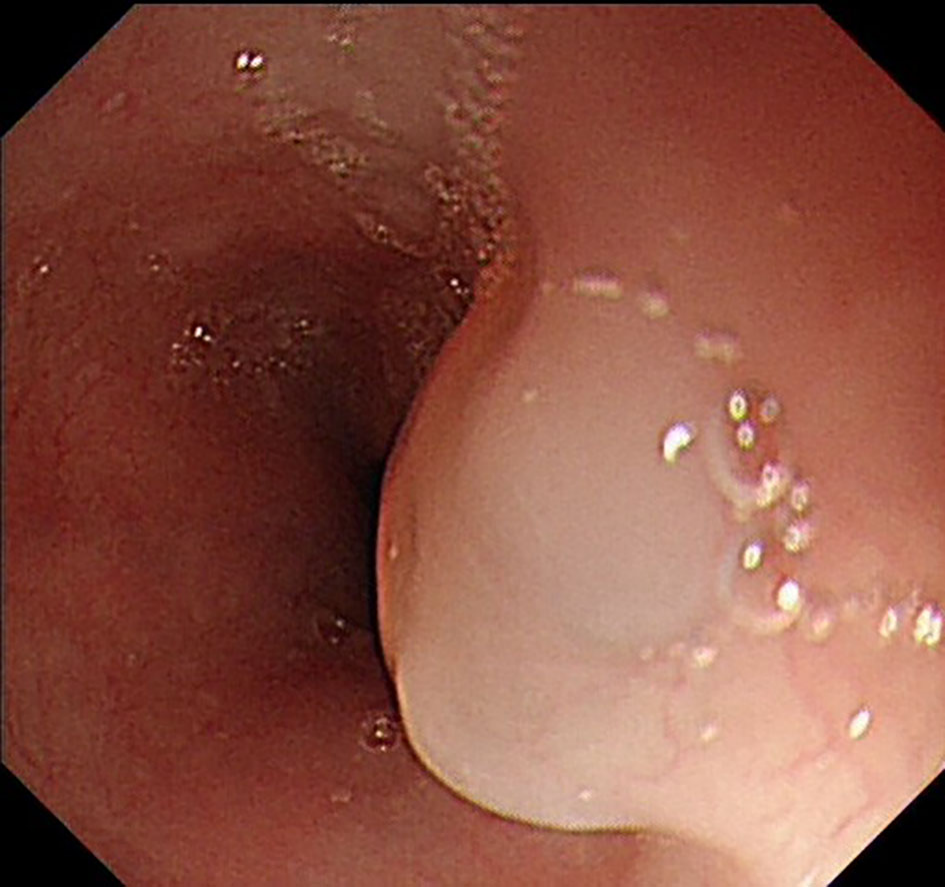
Endoscopic appearance of lymphangioma in the esophagus.

**Figure 3:**
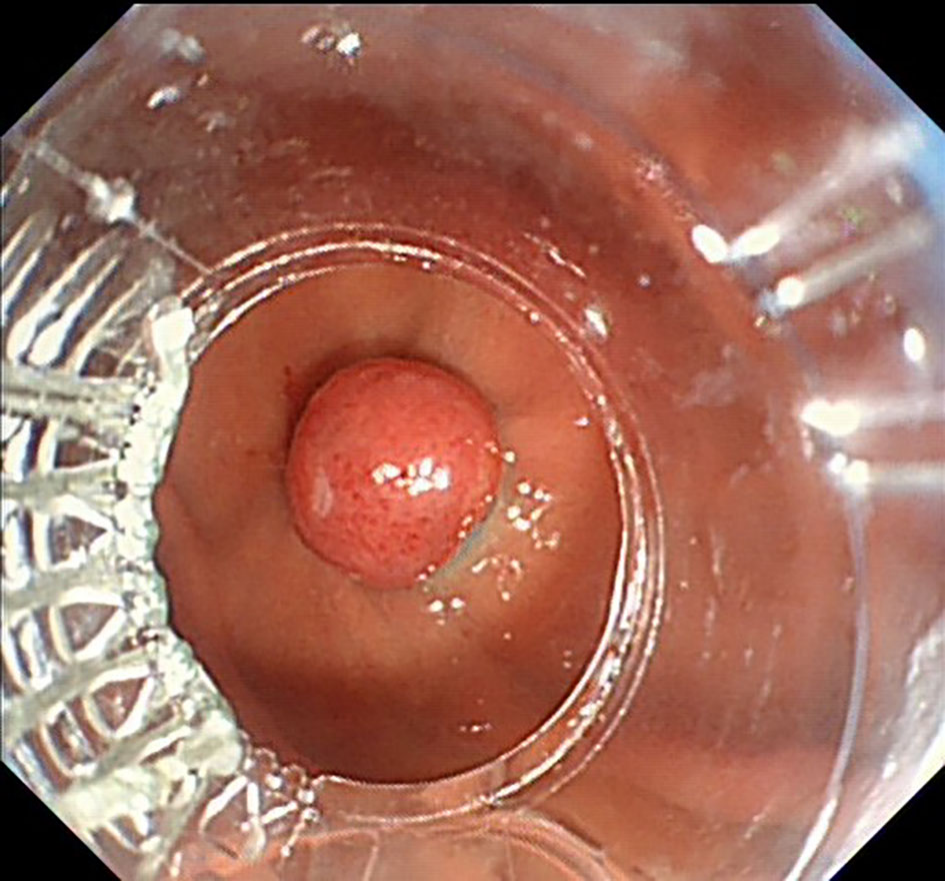
Nodular lymphangioma in the gastric fundus.

**Figure 4:**
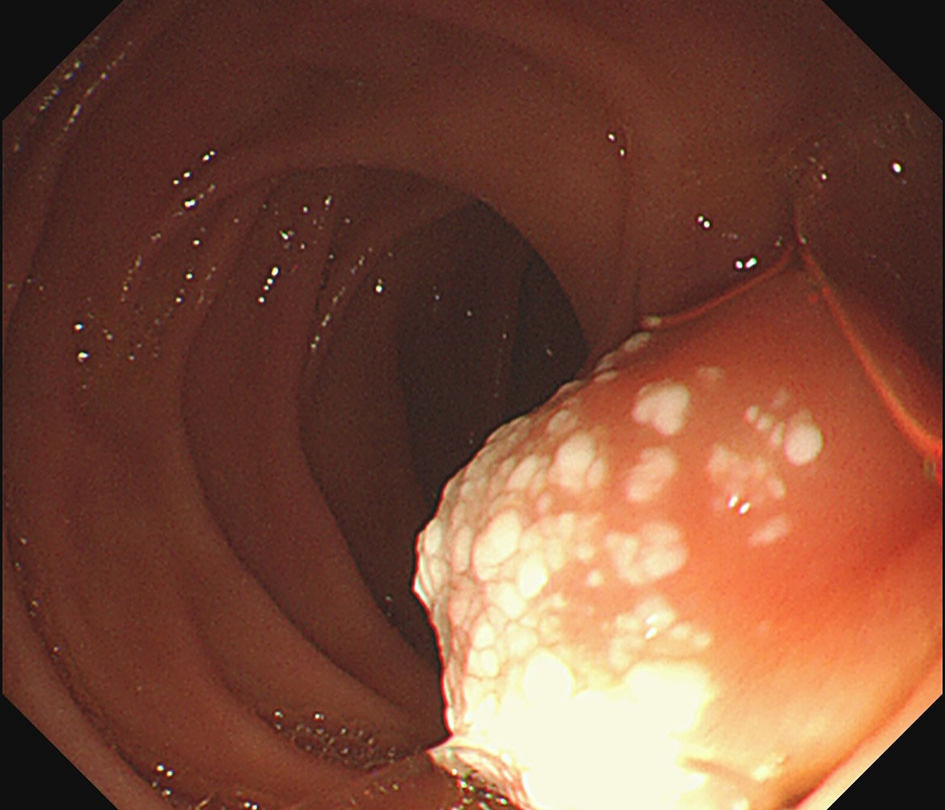
Mucosal prominence with white spots was the endoscopic appearance of the duodenal lesion.

**Figure 5:**
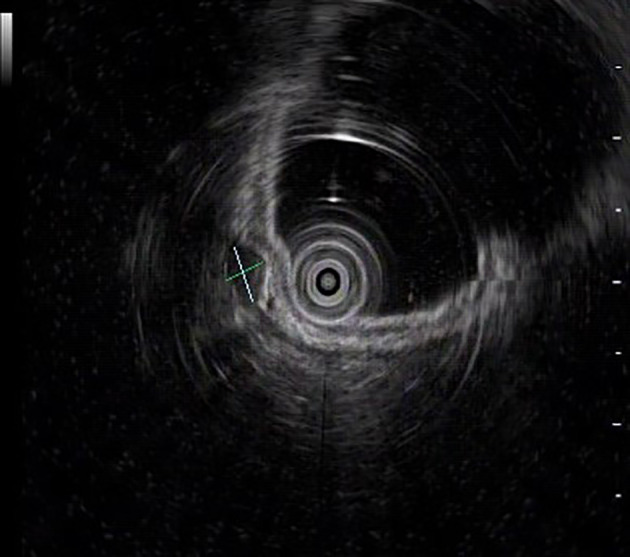
EUS image shows the gastric lesion with hypoechogenicity.

**Figure 6:**
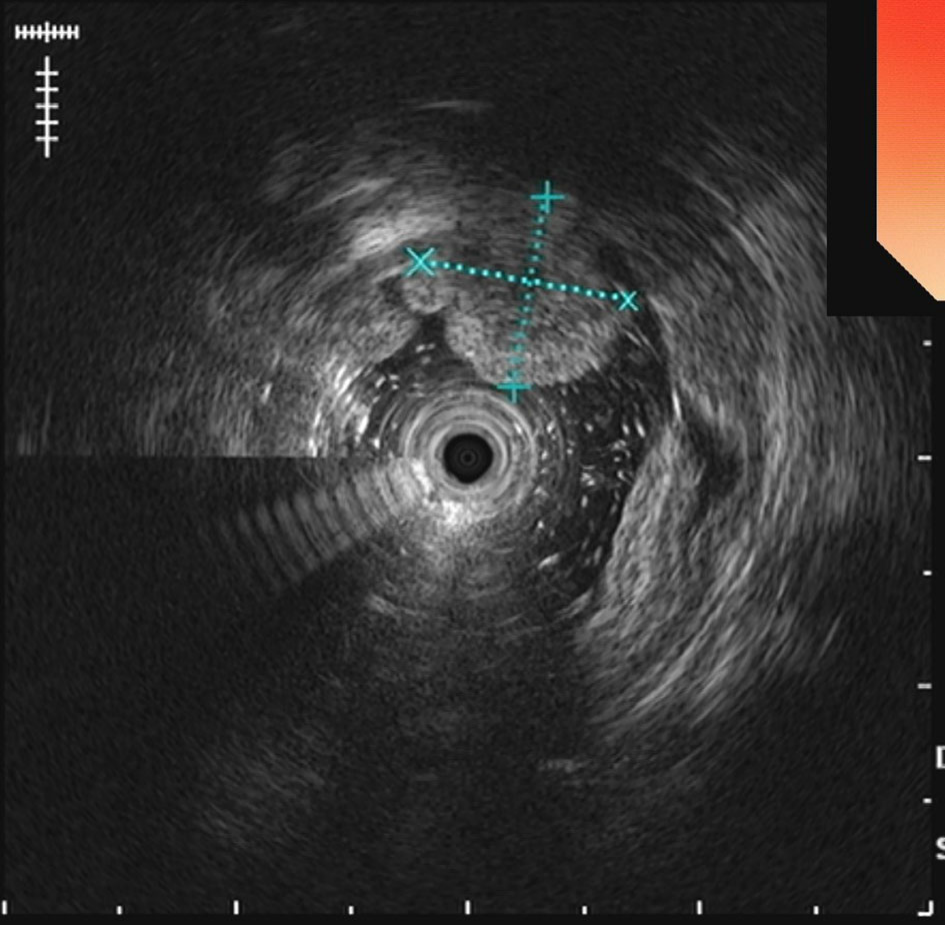
EUS examination demonstrates duodenal lymphangioma with hyperechogenicity.

**Figure 7:**
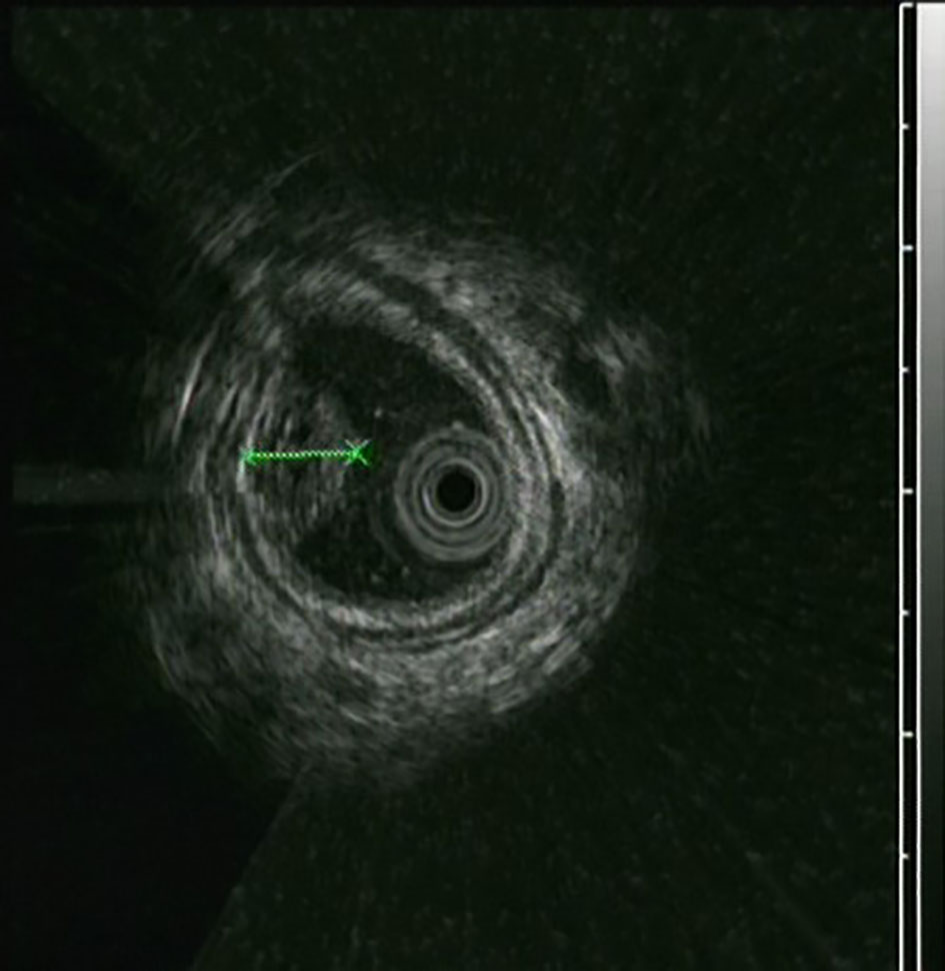
The esophageal lymphangioma was characterized by a heterogeneous echo.

The UGL was unique in 23 patients (95.80%), whereas 1 patient had more than one UGL. 25 lesions found in 24 patients were diagnosed by the endoscope and/or endoscopic ultrasonography before operation (**Figure 1**). Endoscopic appearance typically appears as dilated lymphatic channels beneath the surface epithelium of the protrude mucosal or sub-mucosal lesion (**Figures 2**, **3**, **4**). As related to the ultrasound features of the lesions, their size ranged from 2.2 to 40 mm with a median size of 7.8 mm. The most common site was esophagus (n=14; 58.33%) and duodenum (n=9, 37.50%), followed by stomach (n=1, 4.20%). As for esophageal lesions, they were located 17 to 35 cm distal to the incisor. At pre-operative EUS, 5 (20.83%) originated from the mucosal layer, 13 (54.17%) from the submucosal layer, and 6 (25.00%) from the muscular mucosa layer. Of the 24 patients who underwent EUS, 14(58.33%) patients showed hypoechogenicity (**Figure 5**); 4 (16.67%) patients showed higher echo (**Figure 6**), 5 (20.83%) showed slightly mixed echogenicity (**Figure 7**), and 1 (4.17%) showed equal echogenicity. Of the 20 patients who underwent CT scans, lesions that presented as uneven density were shown in different locations in 9 (37.5%) patients, whereas CT was normal in 11 (45.8%) patients. Imaging data are shown in **Table 2**.

**Table 2:** Endoscopic characteristics.

Patients	N (%)
**Number of lesions**	25
Single	23 (95.83%)
Multiple	1 (4.17%)
**Locations**	
**Esophagus**	14 (58.33%)
Upper esophagus	6 (25.00%)
Middle esophagus	5 (20.83%)
Lower esophagus **Duodenum**	3 (12.50%) 9(37.50%)
Duodenum bulb	1 (4.17%)
duodenum descending part	8 (33.33%)
**Stomach**	1 (4.17%)
Size of lesions mean±SD(range)/mm	7.83±7.76 ( 2.20-40.10)
≤5mm	9 (37.50%)
>5,≤10mm	13 (54.16%)
>10mm	2 (8.33%)
**Origin based on preoperative EUS**	
Mucosa	5 (20.83%)
Submucous	13 (54.17%)
Muscularis mucosa	6 (25.00%)
**Echogenicity based on preoperative EUS**	
Hypo	14(58.33%)
Hyper	4 (16.67%)
Equal	1 (4.17%)
Mixed	5 (20.83%)
**CT scan**	
Yes	9 (37.50%)
No	11 (45.83%)
Unexamined	4 (16.67%)
**Endoscopic treatment**	
EMR	22 (91.67%)
ESD	2 (8.33%)

### Treatment and pathological manifestations

All lesions were successfully resected endoscopically including 23 (91.67%) by endoscopic mucosal resection (EMR) and 2 (8.33%) by endoscopic submucosal dissection (ESD) (**Table 2**). Histologic examination revealed that the lesions contained many irregularly dilated lymphatic vessels, lined with flattened endothelial cells without atypia, and with abundant lymphoid tissue. All those typical findings confirmed the initial diagnosis of lymphangioma in all patients. During and after operation, adverse events occurred in 3 (1 with active gastrointestinal bleeding during the operation and 2 with high fever after wards). All patients recovered.

### Follow−up and recurrence

After the follow-up period ranged from 13 to 92 months (median 43 months), 1 patient was lost to follow-up because of the long period, and 23 survived to whom the last visit was used as the endpoint of follow-up. They all received gastroduodenoscopy and all patients were in good health, and neither recurrence nor death was observed.

## Discussion

As most benign congenital malformations, lymphangiomas most commonly occur in childhood in head, neck, axilla, and mediastinum (9). Only a minority of cases are observed in adult patients and are diagnostically challenging. In addition, lymphangiomas have been extremely rarely reported in the upper gastrointestinal tract, including the esophagus, stomach, and duodenum (7, 8). Esophageal lymphangioma was first reported by Watson-Williams in 1934, and Brady and Milligan were the first persons to diagnose it by endoscopy (14). Possible etiologies are malformation of lymphatic vessels and the obstruction of lymphatic flow by inflammation or injury (15). And the acquired failure of lymphatic channels is more likely associated with the adult manifestations, possibly related to inflammatory conditions or physical trauma such as those result from surgical or radiation therapies (16). While the congenital and acquired causes are not mutually exclusive, a congenital impairment in communication between mesenchymal slits and the venous system may put patients at greater risk of drainage blocking in response to trauma (16, 17). Except for lymphangiectasia was histologically confirmed in one patient seven months before the endoscopic resection of the lymphangioma, no precipitating etiology could be identified in the current adult cases described in our research. In addition, there was no obvious connection between UGL and family tumor history in our study.

To date, only 75 UGLs have been described in English and Chinese language publications since 1934 based on our review, including 31 cases of esophageal lymphangioma, 27 cases of gastric lymphangioma, and 17 cases of duodenal lymphangioma (5, 18–21). Considering almost half of those cases reported later than 2000, it suggests increased use of upper endoscopy and rising awareness of endoscopists on upper gastrointestinal lesions (18, 19, 21). To the best of our knowledge, the present study including 24 patients is the largest scale of research referring to this rare clinical entity in the upper gastrointestinal tract. Cheng et al. (18) found that the esophageal tumors most frequently locate in the distal esophagus, while in Chinese patients, esophageal lymphangioma showed a predilection of upper- and middle-esophagus location. The former study has been confirmed in our research consisting of 6 patients with upper-esophageal lesions and 5 patients with middle-esophageal lesions in all 14 Chinese patients with esophageal lymphangiomas. For duodenal lymphangiomas, the most common site was the duodenum descending part (21), which was also shown in our study. Compared to other gastric parts, previous studies reported that the most common location was the gastric body and antrum (19, 22, 23). Some authors have suggested that it is slightly more common in males than in females compared to our study, but no sex predominance has been confirmed until now (5).

The clinical presentation of these lesions depends on their size and location, and symptoms were diverse regardless of the age of presentation and sites. As most of these UGLs were detected incidentally, previous studies also reported that UGLs were associated more frequently (45%) with non-specific signs and symptoms such as vomiting, dyspepsia, abdominal pain, or were incidental findings (24, 25). And these non-specific signs and symptoms were consistent with our results.

An accurate preoperative diagnosis of UGLs, especially in an adult patient is uncommon owing to its rarity and diverse clinical and radiological features (16, 18). The imaging examination such as computed tomography seems not very helpful in the diagnosis of UGLs. In recent years, with the wide application of EUS which could provide valuable information, EUS became one of the most important diagnostic modalities for vascular tumors arising from the gastrointestinal tract (11, 24). However, the EUS features of gastric lymphangioma are not well demonstrated, possibly owing to its rarity (26). In endoscopy, most UGLs are recognized as polyps, and EUS is used to confirm the size and origin of the lesion (27). The classical characteristics of UGLs under EUS manifest a honeycomb- or grid-like multi-microcystic echo pattern and the lesion may involve lamina propria and submucosal layer. Sometimes, the echo pattern varies according to the size of dilated lymphatic vessels (2, 7, 18). In our experience, endoscopic appearance typically appears as dilated lymphatic channels beneath the surface epithelium of the protrude mucosal or sub-mucosal lesion. We find EUS an excellent initial diagnostic tool, and the presence of a honeycomb-like or grid-like mass with a heterogeneous echo pattern and a clear boundary between the lesion and the muscularis propria layer could be helpful for the primary diagnosis of UGL. As a result, EUS is recommended to all patients with UGLs before final endoscopic or surgery resection.

According to the previous research, complete en bloc resections usually contribute to excellent outcomes and prognoses for patients (28, 29). Many studies have illustrated that endoscopic resection is suitable for smaller lymphangiomas, while laparoscopic surgery is the recommended management for larger tumor involving the muscularis propria or the lesion is suspected malignant (11, 20, 25). In our study, all UGLs were successfully resected endoscopically including 22 (91.67%) by EMR and 2 (8.33%) by ESD. In addition to adverse events occurred in 3 patients who finally recovered, there were no other cases of recurrence during the follow-up period. As a truly minimally invasive technique, endoscopic treatment preserves the integrity of the anatomy and the original function of the organ (30). Hence, we recommend endoscopic resection both for yielding a final histological diagnosis and to prevent them from growing too large for endoscopic management.

It is extremely challenging for the diagnosis of relatively small gastrointestinal lymphangiomas, and histological examination is essential for a definitive diagnosis (26).Margaret et al. reported that it was hard to distinguish lymphangioma because of the histologic overlap with lymphangiectasia of the gastrointestinal mucosa (31). Compared with lymphangiectasia, he demonstrated the most reliable histologic features of lymphangioma as the presence of smooth muscle surrounding the lymphatic spaces and complete circumferential lining of spaces by endothelial-type cells (31, 32). Other types of vascular tumors include haemangiolymphangioma and haemangioma (33). Haemangiolymphangiomas are masses with mucosal and submucosal proliferations of capillary-type blood vessels, and of lymphatic-type vessels. And the cavity contained red blood cells or lymphatic fluid (34). While haemangiomas consist of clustered vascular hyperplasia in the submucosa, lumen irregularity, partial dilation (12). Other studies concluded that the typical features of lymphangiomas are the presence of alternating lymphoid tissue, lymphatic space, and chylous or serous fluid within an irregularly dilated lymphatic channel (16, 20, 25). The histological analysis of the resected mass from our patient provided a clear result for this differential diagnosis that the lesions contained many irregularly dilated lymphatic vessels, lined with flattened endothelial cells without atypia, and with abundant lymphoid tissue.

In conclusion, there were no typical signs of UGL for its clinical manifestations vary in both location and size. EUS has important clinical value for the primary diagnosis lymphangioma in the upper gastrointestinal tract. Because of the endoscopic resection’s low technical complexity and complications for managements of patients with UGLs, this study suggests that this method should be considered as a minimally invasive, safe, feasible, and effective therapeutic option comparing to laparoscopic surgeries. Further studies are needed to confirm our findings.

## Data availability statement

The raw data supporting the conclusions of this article will be made available by the authors, without undue reservation.

## Ethics statement

The studies involving human participants were reviewed and approved by The Ethics Committee of The First Affiliated Hospital of Zhengzhou University. The patients/participants provided their written informed consent to participate in this study.

## Author contributions

ZS, KL, DL, BL: Guarantors of the integrity of the entire study. BL, ZS, XH and QT: Study concepts. ZS, QT and ML: Study design. HY, JZ and YZ: Literature research. ZS, QT and KL: Data acquisition. QT and ML: Data analysis and statistical analysis. ZS, XH and KL: Manuscript preparation. DLL and JZ: Manuscript editing. BL and DL: Manuscript review. All authors contributed to the article and approved the submitted version.

## Funding

This research was supported by the Key R&D Program of Henan Province (No. 222102310038).

## Conflict of interest

The authors declare that the research was conducted in the absence of any commercial or financial relationships that could be construed as a potential conflict of interest.

## Publisher’s note

All claims expressed in this article are solely those of the authors and do not necessarily represent those of their affiliated organizations, or those of the publisher, the editors and the reviewers. Any product that may be evaluated in this article, or claim that may be made by its manufacturer, is not guaranteed or endorsed by the publisher.

## References

[1] Zhou Q Zheng JW Mai HM Luo QF Fan XD Su LX . Treatment guidelines of lymphatic malformations of the head and neck. Oral Oncol (2011) 47(12):1105–9. doi: 10.1016/j.oraloncology.2011.08.001 21906990

[2] Arashiro M Satoh K Osawa H Yoshizawa M Nakano H Ajibe H . Endoscopic submucosal dissection of esophageal lymphangioma: A case report with a review of the literature. Clin J Gastroenterol (2010) 3(3):140–3. doi: 10.1007/s12328-010-0150-4 26190120

[3] Kennedy TL . Cystic hygroma-lymphangioma: A rare and still unclear entity. Laryngoscope (1989) 99(10 Pt 2 Suppl 49):1–10. doi: 10.1288/00005537-198910001-00001 2677565

[4] Hancock BJ St-Vil D Luks FI Di Lorenzo M Blanchard H . Complications of lymphangiomas in children. J Pediatr Surg (1992) 27(2):220–4. doi: 10.1016/0022-3468(92)90316-Y 1564622

[5] Rana A Katzman PJ Pegoli W Qualia C . An unusual cause of abdominal pain: duodenal cystic lymphangioma. Gastroenterol Hepatol (N Y) (2013) 9(3):192–5. PMC3745212 23961273

[6] Cui J Huang LY Lin SJ Yi LZ Wu CR Zhang B . Small intestinal vascular malformation bleeding: A case report with imaging findings. World J Gastroenterol (2014) 20(38):14076–8. doi: 10.3748/wjg.v20.i38.14076 PMC4194596 25320550

[7] Gale EAM . Endoscopic submucosal dissection of a giant esophageal lymphangioma. Endoscopy (2018) 50(07):E181–E3. doi: 10.1055/a-0605-2443 29742767

[8] Sriram P Weise C Seitz U Brand B . Lymphangioma of the major duodenal papilla presenting as acute pancreatitis: Treatment by endoscopic snare papillectomy. J Gastrointest Endoscopy (2000) 51(6):733–6. doi: 10.1067/mge.2000.106111 10840315

[9] Ali HA Zeriouh B Bouzayan L Jabi R Bouziane M . Giant cystic lymphangioma of the stomach: A case report. Ann Med Surg (Lond) (2021) 61:8–12. doi: 10.1016/j.amsu.2020.12.010 33363719 PMC7750175

[10] Saers T Parusel M Brockmann M Krakamp B . Lymphangioma of the esophagus. Gastrointest Endosc (2005) 62(1):181–4. doi: 10.1016/S0016-5107(04)02844-5 15990849

[11] Zhao ZF Kuang L Zhang N Ma SR Yang Z Han X . Endoscopic diagnosis and treatment of esophageal cavernous lymphangioma. Surg Laparosc Endosc Percutan Tech (2013) 23(3):299–302. doi: 10.1097/SLE.0b013e31828b8810 23751996

[12] Hu PF Chen H Wang XH Wang WJ Su N Shi B . Small intestinal hemangioma: Endoscopic or surgical intervention? a case report and review of literature. World J Gastrointest Oncol (2018) 10(12):516–21. doi: 10.4251/wjgo.v10.i12.516 PMC6304305 30595805

[13] Matsuda T Hiki N Nunobe S Aikou S Hirasawa T Yamamoto Y . Feasibility of laparoscopic and endoscopic cooperative surgery for gastric submucosal tumors (with video). Gastrointest Endosc (2016) 84(1):47–52. doi: 10.1016/j.gie.2015.11.040 26684599

[14] Brady PG Milligan FD. . Lymphangioma of the esophagus–diagnosis by endoscopic biopsy. Am J Dig Dis (1973) 18(5):423–5. doi: 10.1007/BF01071994 4701042

[15] Perkins JA Manning SC Tempero RM Cunningham MJ Edmonds JL Jr. Hoffer FA . Lymphatic malformations: current cellular and clinical investigations. Otolaryngol Head Neck Surg (2010) 142(6):789–94. doi: 10.1016/j.otohns.2010.02.025 20493347

[16] Kang BH Hur H Joung YS Kim DK Kim YB Ahn CW . Giant mesenteric cystic lymphangioma originating from the lesser omentum in the abdominal cavity. J Gastric Cancer (2011) 11(4):243–7. doi: 10.5230/jgc.2011.11.4.243 PMC3273697 22324018

[17] van Oudheusden TR Nienhuijs SW Demeyere TB Luyer MD de Hingh IH . Giant cystic lymphangioma originating from the lesser curvature of the stomach. World J Gastrointest Surg (2013) 5(10):264–7. doi: 10.4240/wjgs.v5.i10.264 PMC3812440 24179624

[18] Cheng Y Zhou X Xu K Huang Q . Esophageal lymphangioma: A case report and review of literature. BMC Gastroenterol (2019) 19(1):107. doi: 10.1186/s12876-019-1026-9 31242868 PMC6595566

[19] Ishikawa N Fuchigami T Kikuchi Y Kobayashi H Sakai Y Nakanishi M . EUS for gastric lymphangioma. Gastrointest Endosc (2000) 52(6):798–800. doi: 10.1067/mge.2000.108292 11115926

[20] Haibin Z Lingling W Lexing Z Xumin B Yingyu W Jianfeng Y . Clinicopathological characteristics and prognosis of gastrointestinal vascular tumours. Sci Rep (2021) 11(1):16062. doi: 10.1038/s41598-021-94821-1 34373472 PMC8352902

[21] Qian W Weishun C . Duodenal lymphangioma: one case report and literature review. Chin J Gastroenterol Hepatol (2019) 28(12):4.

[22] Matsushita A Yuasa N Miyake H Nagai H Nagao T Fujino M . Gastric lymphangioma coexisting with mucosal gastric cancer: A rare case report. Clin J Gastroenterol (2020) 13(1):46–9. doi: 10.1007/s12328-019-01013-6 31264079

[23] Bai K Dai Y Jiang C Lin S Wang G . Gastric lymphangioma: A case report and review of literature. BMC Gastroenterol (2022) 22(1):407. doi: 10.1186/s12876-022-02431-6 36058923 PMC9441034

[24] Handra-Luca A Montgomery E . Vascular malformations and hemangiolymphangiomas of the gastrointestinal tract: morphological features and clinical impact. Int J Clin Exp Pathol (2011) 4(5):430–43. PMC3127065 21738815

[25] Luo D Ye L Wu W Zheng H Mao X . Huge lymphangioma of the esophagus resected by endoscopic piecemeal mucosal resection. Case Rep Med (2017) 2017:5747560. doi: 10.1155/2017/5747560 28408932 PMC5376932

[26] Nayak M Purkait S Sasmal PK Singh PK . Cystic lymphangioma of the stomach with marked reactive changes: A rare cause of gastric outlet obstruction in adult. BMJ Case Rep (2020) 13(7):e233582. doi: 10.1136/bcr-2019-233582 PMC7348654 32641314

[27] Tanaka Y Fujii S Kusaka T Kokuryu H . Effective use of EUS for diagnosing a jejunal lymphangioma accompanied with hemorrhage. Gastrointest Endosc (2020) 91(1):199–200. doi: 10.1016/j.gie.2019.08.009 31442393

[28] Ghatak S Ray S Sanyal S Sonar PK Khamrui S Basu K . An unusual cause of acute abdomen in adults: giant cystic lymphangioma of the pancreatic head. A Clin Case Literature Rev JOP (2011) 12(3):266–70. doi: 10.6092/1590-8577/3295 21546706

[29] Zhuang K Jiang X Huang S . A rare, giant, cystic, and cavernous lymphangioma originated from the stomach in A young woman. J Gastrointest Surg (2019) 23(6):1271–3. doi: 10.1007/s11605-018-3877-8 30039446

[30] Matsushita M Ueshima T Nishimon S Takahashi M Asayama T Shibatani N . Unroofing technique: effective "incomplete" endoscopic resection of large esophageal lymphangiomas. Endoscopy (2020) 52(7):621. doi: 10.1055/a-1157-8888 32580229

[31] Lawless ME Lloyd KA Swanson PE Upton MP Yeh MM . Lymphangiomatous lesions of the gastrointestinal tract: A clinicopathologic study and comparison between adults and children. Am J Clin Pathol (2015) 144(4):563–9. doi: 10.1309/AJCPO8TW6EMAJSRP 26386077

[32] Min M Liu Y . Lymphangioma of the esophagus. Am J Gastroenterol (2018) 113(7):936. doi: 10.1038/s41395-018-0071-2 29867174

[33] Tseng JJ Chou MM Ho ES . Fetal axillary hemangiolymphangioma with secondary intralesional bleeding: serial ultrasound findings. Ultrasound Obstet Gynecol (2002) 19(4):403–6. doi: 10.1046/j.1469-0705.2002.00633.x 11952973

[34] Gomez-Galan S Mosquera-Paz MS Ceballos J Cifuentes-Grillo PA Gutierrez-Soriano L . Duodenal hemangiolymphangioma presenting as chronic anemia: A case report. BMC Res Notes (2016) 9(1):426. doi: 10.1186/s13104-016-2214-0 27581369 PMC5007800

